# Ovarian Cancer Stem Cells: Mechanisms of Progression and Therapeutic Strategies

**DOI:** 10.32604/or.2026.083359

**Published:** 2026-08-13

**Authors:** Jie Wu, Zhewei Zhang, Kit Ying Chan, Tat San Lau, Chi Chiu Wang

**Affiliations:** 1 Department of Obstetrics and Gynaecology, Faculty of Medicine, The Chinese University of Hong Kong, Prince of Wales Hospital, Shatin, Hong Kong, China; 2 Li Ka Shing Institute of Health Sciences, Faculty of Medicine, The Chinese University of Hong Kong, Shatin, Hong Kong, China; 3 School of Biomedical Sciences, The Chinese University of Hong Kong, Hong Kong, China

**Keywords:** Ovarian cancer, cancer stem cells, chemoresistance, tumor microenvironment, targeted therapy

## Abstract

Ovarian cancer is the most lethal gynecological malignancy, with most patients diagnosed at an advanced stage and eventually relapsed after post-platinum-taxane chemotherapy. High intratumoral heterogeneity, extensive peritoneal dissemination, and acquired chemoresistance continue to restrict the clinical benefits of current therapeutic strategies. Increasing evidence indicates that ovarian cancer stem cells (OCSCs), a rare but highly plastic subpopulation characterized by self-renewal, multilineage differentiation, quiescence, tumor-initiating capacity, and intrinsic stress tolerance, play pivotal roles in tumor initiation, metastasis, recurrence, and therapeutic resistance. In this review, we systematically summarize current knowledge regarding the identification and functional characterization of OCSCs and discuss their clinical relevance to disease progression and poor prognosis. We then delineate the multifaceted mechanisms by which OCSCs drive ovarian cancer progression, with particular emphasis on dysregulated stemness-associated signaling networks, adaptive cellular plasticity, metabolic and stress-response programs, and dynamic interactions with the tumor microenvironment. Furthermore, we review emerging therapeutic strategies aimed at eradicating OCSCs and discuss key barriers that hinder their clinical translation. A deeper understanding of OCSC biology may facilitate the development of precise, combination-based strategies to overcome recurrence, reverse therapeutic resistance, and improve long-term outcomes in ovarian cancer patients.

## 1 Introduction

As the most lethal gynecological malignancy, ovarian cancer (OC) presents major clinical challenges characterized by frequent late-stage diagnosis, high recurrence rates, and poor prognosis [1,2,3]. Driven by high tumor heterogeneity and extensive intraperitoneal metastasis, the five-year survival rate for patients with advanced-stage disease has long stagnation below 30% [1,4,5]. Although initial cytoreductive surgery combined with chemotherapy can induce clinical remission, the emergence of chemoresistance leads to relapse in the vast majority of patients [6,7,8,9]. Among relapsed cases, the median overall survival (OS) for platinum-resistant cohorts is a mere 9 to 16 months [5,10]. Even emerging targeted therapies, such as bevacizumab and PARP inhibitors, and immunotherapies including immune checkpoint inhibitors (ICIs) offer limited clinical benefits. Their efficacy is largely constrained by the immunosuppressive and low-immunogenic tumor microenvironment (TME), with clinical trials revealing an ICI response rate of only 10–15% [11,12].

In-depth studies reveal that initiation, metastasis, and recurrence of OC are not random events; rather, accumulating evidence identifies ovarian cancer stem cells (OCSCs) as the primary driving force behind these processes [13,14,15]. Initially isolated from patient ascites and demonstrating robust tumorigenic capacity, OCSCs are endowed with stemness, plasticity, and innate chemoresistance. These traits enable them to survive cytotoxic therapies and enter a state of dormancy, eventually mediating tumor regeneration and distant metastasis under favorable conditions [1,16,17,18]. Furthermore, the TME plays a crucial supportive role in maintaining OCSC stemness and facilitating their phenotypic transitions [19,20].

Given the pivotal role of OCSCs in tumor relapse and drug resistance, deciphering their biological behaviors and regulatory networks is essential to overcome the chemoresistance bottleneck [21,22,23]. This review systematically outlines the core mechanisms of OCSCs in OC progression and summarizes recent advances in OCSC-targeted therapies, aiming to provide a theoretical foundation for enhancing patient outcomes.

## 2 Cancer Stem Cells in Ovarian TME

To understand how OCSCs contribute to ovarian cancer progression, it is necessary to understand how this cell population is defined. In ovarian cancer, OCSCs are best regarded as a functionally defined stem-like state rather than a uniform cell lineage with a single established origin [24,25]. Although the cellular origin of ovarian carcinoma itself is histotype-dependent and remains to be determined by pathological professionals, the OCSC-like properties within established tumors appears to be highly dynamic [26,27]. These properties may be inherited from transformed progenitor-like cells, enriched through the selection of pre-existing stem-like subclones, or reacquired by more differentiated tumor cells under the influence of EMT/MET plasticity, hypoxia, inflammatory cytokines, stromal interactions, and therapeutic pressure [24,28,29]. Therefore, phenotypic markers are best viewed as tools for enriching and tracking candidate OCSC-like populations, rather than as stand-alone determinants of OCSC identity [30]. OCSC-like populations are commonly recognized by integrating a combination of functional features such as self-renewal, multilineage differentiation, tumor-initiating capacity, and resistance to therapeutic stress.

### 2.1 Identification and Characterization of OCSCs Based on Phenotypic and Functional Markers

Accurate enrichment and characterization of OCSC-like populations are prerequisites for analyzing their biological functions and developing targeted therapies. At present, immunosorting based on cell surface antigens or intracellular enzymatic activity remains the most widely used approach to isolate candidate OCSC-enriched fractions [1,2,19,21]. These markers are well studied in defining subpopulations and facilitated mechanistic and translational studies. However, because marker expressions can be observed in non-OCSC tumor cells or normal stem/progenitor cells, which may also change under microenvironmental or therapeutic pressure, thereby marker-based enrichment should be interpreted together with supplementary functional assays [31].

Currently, the most widely recognized markers of OCSC-like populations include (Table 1).

**Table 1:** OCSC-associated markers: key functions and clinical correlations.

Marker	Function	Clinical Correlation	Ref.
CD133	Maintains undifferentiated state; marks tumor-initiating/chemoresistant ovarian cancer (OC) cells; involved in differentiation/EMT-related interactions, autophagy, adhesion/metastasis	Higher in OC vs. normal; correlates with ascites, advanced stage, chemoresistance; associated with platinum resistance, CNS metastasis risk, poor prognosis; independent predictor of shorter DFS	[1,2,3,21,32,33,34,35,36,37,38]
CD44	Adhesion/migration signaling; promotes stemness, chemoresistance, metastasis (HA receptor); CD44^+^/CD24^−^ subset has stem-like traits	CD44^+^ OCSCs Chemoresistant and linked to poor prognosis; in stage IIIB–IVA serous EOC (n = 96), CD44 positivity correlated with shorter DFS/OS	[2,39,40,41,42,43,44,45,46,47,48]
ALDH1	Intracellular enzyme; OCSC functional marker; supports stemness & chemoresistance via detox; regulated by Wnt/β-catenin, MUC1-C/ERK, NF-κB; ALDH^+^/CD133^+^ highly tumorigenic	Overexpression/activity associated with lower OS, advanced FIGO stage, metastasis, lymphatic invasion; ALDH1A1^+^ tumors more often platinum-insensitive (17% vs. 6%)	[2,22,49,50,51,52,53,54,55]
CD117	SCF receptor tyrosine kinase; maintains plasticity/stem-like phenotype (RAS/ERK, PI3K, JAK/STAT, Wnt, Notch); OC-derived CD117^+^ cells show self-renewal/differentiation; promotes cisplatin/paclitaxel resistance	Meta-analysis: associated with age, FIGO stage, histotype, differentiation; high CD117 link to worse OS (HR = 1.39)	[56,57,58,59]
LGR5	Overexpression promotes proliferation/metastasis/EMT via Notch1; LGR5 downregulation inhibits proliferation & *in vitro* tumorigenicity and shifts EMT markers	High LGR5 expression in primary EOC correlates with advanced FIGO stage, higher grade, and poorer OS; aberrant LGR5 expression is also associated with age, histological type, and distant metastasis.	[60,61,62,63,64]
CD24	GPI-anchored; activates PI3K/Akt, NF-κB, ERK → EMT/invasion/cisplatin resistance; enriches stemness genes & STAT3 activation; CD24^+^ cells form xenografts; matched CD24^−^ cells do not in this model	Generally associated with advanced stage & poor prognosis; independent prognostic marker; higher expression → poorer survival; CD24^+^ more chemoresistant/quiescent with self-renewal/differentiation capacity	[2,65,66,67,68]
EpCAM	Epithelial adhesion molecule; in OC, EpCAM^+^ cells show stronger tumor initiation, self-renewal, multilineage differentiation	Higher in tumors from chemoresistant patients; associated with poor prognosis	[69,70,71]

CSC, cancer stem cell; OCSC, ovarian cancer stem cell; EOC, epithelial ovarian cancer; EMT, epithelial–mesenchymal transition; FIGO, international federation of gynecology and obstetrics; OS, overall survival; PFS, progression-free survival; IHC, immunohistochemistry; TMA, tissue microarray.

#### 2.1.1 CD133 (Prominin-1)

CD133 is a pentaspan transmembrane glycoprotein that maintains an undifferentiated state and induces tumors in immunodeficient mice [32,33]. Initially identified in hematopoietic and neural stem cells [34], it influences OC progression by participating in primitive cell differentiation, epithelial-mesenchymal interactions (EMT), and autophagy, while regulating metastatic adhesion [35]. CD133^+^ OC cells exhibit enhanced tumor-initiating capacity and chemoresistance [1,2,21,34]. Ferrandina et al. reported higher CD133-1/2 epitope abundance in ovarian tumors than in benign/normal tissues, with CD133^+^ cells showing superior clonogenicity and proliferation [36]. CD133 expression positively correlates with well-differentiated serous carcinoma, ascites, advanced stage, and chemoresistance [36]. Furthermore, primary tumor CD133 expression correlates with platinum resistance, elevated CNS metastasis risk, and poor prognosis [37]. Multivariate analysis of 400 OC samples identified CD133 as an independent predictor of shortened DFS [38].

#### 2.1.2 CD44

Expressed on various mammalian cells [39], CD44 mediates cell adhesion, migration, and signaling. It enhances cytoskeletal rearrangement, altering cell stiffness to drive migration, stemness, chemoresistance, and immunosuppression [40,41]. As a primary hyaluronic acid (HA) receptor, CD44 binds HA to activate Merlin, thereby stabilizing intercellular adhesion and promoting contact inhibition. In other contexts, CD44 interacts with ERM proteins and its intracellular tail can facilitate cell migration and metastasis [42,43,44]. The CD44^+^/CD24^−^ subpopulation in OC possesses stem-like properties linked to metastasis and chemoresistance [2]. CD44^+^ CSCs are chemoresistant and predict poor prognosis [45,46,47]. In 96 patients with stage IIIB-IVA primary serous EOC, 49% expressed CD44, which significantly correlated with shortened DFS (*p* ≤ 0.001) and OS (*p* ≤ 0.001), likely by promoting carboplatin resistance [48].

#### 2.1.3 Aldehyde Dehydrogenase 1 Family Member A1 (ALDH1A1)

ALDH1A1, an intracellular enzyme involved in retinoic acid synthesis, is a recognized CSC marker [2]. Regulated by oncogenic pathways (Wnt/*β*-catenin, MUC1-C/ERK) and NF-*κ*B signaling [49,50], it influences cell differentiation, stemness, tumorigenesis, and resistance [51]. High ALDH1A1 activity correlates with OC stemness and chemoresistance [2], as these cells efficiently scavenge toxic aldehydes to resist chemotherapy [22]. Its role in chemoresistance was first noted in paclitaxel/platinum-resistant cells, in which high ALDH1A1 expression negatively correlated with survival; in xenograft assays, as few as 11 ALDH1A1^+^/CD133^+^ cells were sufficient to initiate tumors [52]. Meta-analyses in ovarian cancer confirmed that ALDH1 overexpression correlates with poorer OS, advanced FIGO stage, and metastasis [53,54], and is also associated with lymphatic invasion (OR = 2.78, *p* = 0.034) [54]. Furthermore, ALDH1A1^+^ tumors are three times more likely to be platinum-insensitive than ALDH1A1^−^ tumors (17% vs. 6%, *p* = 0.04) [55].

#### 2.1.4 CD117 (c-Kit)

CD117 is a transmembrane tyrosine kinase receptor that primarily functions as the receptor for stem cell factor (SCF), participating in the regulation of various biological processes such as cell proliferation, differentiation, migration, apoptosis, and adhesion [56,57]. Studies indicate that CD117 signaling is crucial for maintaining cellular plasticity. During carcinogenesis, activation of CD117 initiates downstream signaling pathways, promoting the formation of stemness or a stem cell-like phenotype, including the RAS/ERK, PI3K, SRC, JAK/STAT, Wnt, and Notch pathways [57]. Indeed, CD117-expressing cells isolated from OC have been demonstrated to exhibit differentiation capacity, self-renewal potential, and stem cell properties [58]. Consistent with these observations, a meta-analysis comprising seven studies of patients with epithelial ovarian cancer (EOC) found that CD117 expression was significantly associated with age, FIGO stage, histological type, and tumor differentiation grade. Moreover, patients with high CD117 expression had significantly poorer overall survival (HR = 1.39, 95% CI 1.03–1.90), whereas those with low CD117 expression did not [59].

#### 2.1.5 Leucine-Rich Repeat-Containing G-Protein Coupled Receptor 5 (LGR5)

LGR5 is a glycoprotein hormone receptor and a key regulator of Wnt signaling. It is expressed in stem cells in multiple tissues and is considered a CSC marker [60]. In colorectal cancer, mouse studies support a functional role for LGR5^+^ cells in tumor maintenance and propagation, consistent with their stem-like properties in primary and metastatic disease [61]. In ovarian cancer, LGR5 overexpression has been observed and is associated with proliferation, metastasis, and EMT via Notch1 signaling [62]. Conversely, LGR5 downregulation inhibits proliferation and reduces *in vitro* tumorigenicity, with increased E-cadherin and decreased N-cadherin and vimentin [63]. Clinically, in 100 primary EOC cases, high LGR5 expression was higher than in normal ovary and benign tumors and correlated with advanced FIGO stage, higher grade, and poorer OS [64]. A tissue microarray study (n = 93) also linked aberrant LGR5 expression with age, histological type, and distant metastasis [60].

#### 2.1.6 CD24

CD24 is a mucin-like cell surface molecule localized to lipid rafts via its glycosylphosphatidylinositol (GPI) anchor [2]. By activating the PI3K/Akt, NF-*κ*B, and ERK signaling pathways, CD24 promotes EMT, a process that plays a critical role in cell colony formation, invasive capacity, and cisplatin resistance [65]. Furthermore, CD24-positive cells express higher mRNA levels of stemness genes, including those encoding neuropeptides, Bmi-1, *β*-catenin, Notch1, Notch4, Oct3/4, and Oct4 [66]. These gene products are involved in regulating various stem cell functions. Burgos-Ojeda et al. demonstrated that the CD24^+^ OCSC subpopulation highly expresses stemness genes (such as NANOG, MYC, and CCND1) and exhibits elevated levels of STAT3 phosphorylation, which promotes the growth and metastasis of ovarian cancer [67]. In addition, animal model experiments have shown that injection of CD24^+^ cells can induce tumor xenograft formation in nude mice, whereas the injection of an equal number of CD24^−^ cells did not produce this effect [66]. Most published studies indicate that CD24 expression is associated with advanced disease stages and poor prognosis. In ovarian cancer patients, CD24 is considered as an independent prognostic marker for survival; among patients undergoing primary surgery, higher CD24 expression is significantly associated with poorer survival rates [68]. Gao et al. found that *in vitro* CD24^+^ cells possess stem cell-like characteristics of a residual quiescent state, exhibiting self-renewal and differentiation capacities, and are more chemoresistant than CD24^−^ cells [66].

#### 2.1.7 Epithelial Cell Adhesion Molecule (EpCAM)

EpCAM, a type I transmembrane glycoprotein widely expressed on the surface of epithelial cells, regulates intercellular adhesion. It is highly expressed in many epithelial-derived tumors and is associated with cancer stem cell properties [69,70]. In ovarian cancer, EpCAM is also considered an important marker for OCSCs. Studies show that EpCAM^+^ OC cells possess stronger tumor-initiating capacity than EpCAM^−^ cells, along with self-renewal and multilineage differentiation capabilities. EpCAM expression levels are elevated in the tumors of chemoresistant patients and are correlated with a poor prognosis [71].

#### 2.1.8 Subtype-Specific and Context-Dependent Features of OCSCs

Ovarian cancer comprises biologically distinct histological subtypes, mainly including high-grade serous, low-grade serous, endometrioid, clear cell, and mucinous carcinomas. Accordingly, OCSC markers and functional traits identified in one ovarian cancer histotype should not be assumed to apply to other histotypes. However, evidence on OCSCs across different histotypes is still limited, and the available data are concentrated mainly in high-grade serous ovarian carcinoma (HGSOC) and clear cell carcinoma. In serous ovarian adenocarcinoma, CD44^+^CD117^+^ spheroid-derived cells have been shown to possess tumor-initiating capacity and chemoresistance. In HGSOC, CD133 and ALDH1 have been examined in paired primary and recurrent tumors, and CD133/ALDH1 co-expression in primary tumors has been associated with poor clinical outcome. In OCCC, ALDH-high cancer stem-like cells have been associated with advanced disease, reduced progression-free survival, lower ROS levels, and increased Nrf2-mediated antioxidant activity. However, studies on endometrioid, mucinous, and low-grade serous carcinomas remains limited and are largely restricted to marker expression analysis, ALDH isozyme analyses, or cell-line-based observations. Therefore, systematic cross-histotype studies using patient-derived models, single-cell approaches, and functional validation are needed before robust subtype-specific OCSC conclusions can be drawn.

The individual or combined use of these markers facilitates the enrichment, identification, and functional study of OCSCs, thereby providing a solid basis for targeted therapies against OCSCs.

### 2.2 Core Characteristics of OCSCs

**Figure 1:**
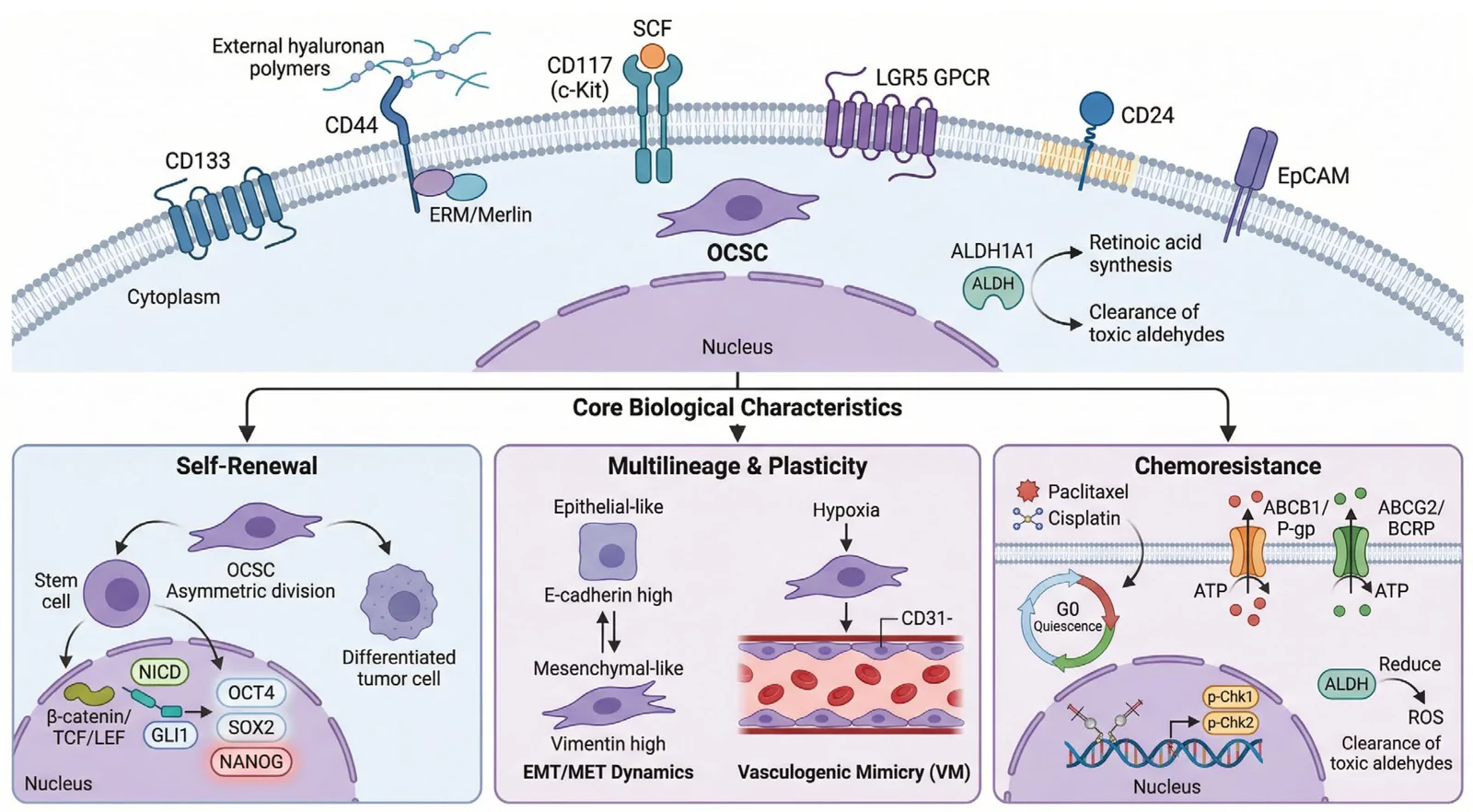
Characteristic markers and core biological functions of OCSCs. The upper panel summarizes representative OCSC-associated markers, including CD133, CD44, CD117, LGR5, CD24, EpCAM, and ALDH1A1, which contribute to OCSC identification and are associated with stemness maintenance, adhesion, cellular plasticity, metastasis, and chemoresistance. The lower panel illustrates the major biological characteristics of OCSCs, including self-renewal, multilineage differentiation plasticity, and chemoresistance. These features are regulated by stemness-related signaling pathways and transcription factors, EMT/MET dynamics, vasculogenic mimicry, cellular quiescence, ABC transporter-mediated drug efflux, enhanced DNA damage repair, ALDH-mediated detoxification, and reactive oxygen species regulation.

As summarized in Fig. 1, OCSCs are characterized by representative surface and functional markers as well as key biological properties, including self-renewal, multilineage differentiation/plasticity, and chemoresistance.

#### 2.2.1 Self-Renewal

Self-renewal is one of the most fundamental characteristics of cancer stem cells. Through symmetric and asymmetric division, they generate new stem cells while simultaneously producing progeny cells capable of further differentiation, thereby maintaining tumor heterogeneity and driving continuous tumor growth [15,72,73,74]. This self-renewal capacity ensures that even if the majority of tumor cells are eradicated by chemotherapeutic agents, a small population of surviving OCSCs can reinitiate tumor proliferation, thus serving as a major driver of tumor recurrence [75,76].

Molecularly, OCSC self-renewal is strictly governed by conserved embryonic signaling pathways, with Wnt, Notch, and Hedgehog forming the core stemness network. In ovarian cancer, Wnt activation inhibits the *β*-catenin destruction complex, driving *β*-catenin nuclear translocation to bind TCF/LEF. This initiates the transcription of stemness genes like LGR5 and AXIN2, maintaining an undifferentiated state [77]. Additionally, Notch3 is highly expressed in ovarian cancer spheroids; its intracellular domain release directly mediates chemoresistance and self-renewal. Specifically inhibiting Notch3 impairs OCSC clonogenicity and induces apoptosis [78]. Moreover, nuclear accumulation of the Hh transcription factor GLI1 positively correlates with CD44 and CD133 expression, driving tumor recurrence [79].

Downstream of these signaling pathways, a series of core pluripotency transcription factors, primarily OCT4, SOX2, and NANOG, form the intrinsic regulatory hub that maintains OCSC self-renewal. These factors are typically expressed in embryonic stem cells but are aberrantly reactivated in high-grade serous ovarian cancer. A classic study by Zhang et al. indicated that the CD44^+^/CD117^+^ subpopulation isolated from ovarian cancer tissues highly expresses OCT4 and NANOG, endowing them with robust tumorigenic capacity and self-renewal potential [80]. More importantly, these transcription factors not only maintain stemness but also directly regulate the expression of ATP-binding cassette (ABC) transporters. This synergistic mechanism enables OCSCs to efficiently pump out chemotherapeutic drugs like paclitaxel and cisplatin while undergoing self-renewal. Consequently, the clinically common minimal residual disease (MRD) becomes difficult to eradicate, ultimately leading to relapse [81].

OCSC self-renewal also depends on supportive niche signals within malignant ascites and the peritoneal microenvironment [82]. Hypoxia and stromal cues from TAMs and CAFs converge on stemness-associated pathways, including Notch and IL-6/JAK2-STAT3, to promote OCSC enrichment and anchorage-independent survival [83,84,85].

#### 2.2.2 Multilineage Differentiation

Beyond sustaining tumor growth, OCSCs undergo asymmetric division to produce the non-tumorigenic bulk of the tumor. This multilineage differentiation drives ovarian cancer heterogeneity, generating diverse subpopulations, such as epithelial, mesenchymal, and vascular endothelial-like phenotypes, that significantly complicate clinical management [80].

The differentiation trajectory of OCSCs is closely linked to epithelial-mesenchymal plasticity, particularly EMT/MET switching in response to microenvironmental cues. During dissemination, OCSCs can adopt a more migratory mesenchymal-like state, whereas metastatic colonization is associated with reacquisition of epithelial proliferative features; Wnt/β-catenin signaling contributes to both stemness maintenance and this phenotypic transition [79,86].

Moreover, the multilineage differentiation potential of OCSCs is not restricted to epithelial or mesenchymal lineages; it also exhibits cross-lineage differentiation capacity, known as vasculogenic mimicry (VM) [87,88]. Under hypoxia, ovarian cancer cells exhibit enhanced VM-associated plasticity through EMT-related reprogramming, and aggressive ovarian tumors can contain tumor cell-lined, non-endothelial channels with red blood cells [88,89]. Importantly, Alvero et al. demonstrated that stem-like ovarian cancer cells can serve as tumor vascular progenitors by forming vessel-like structures and acquiring endothelial markers [87]. Similarly, Tang further showed that ovarian cancer stem-like cells differentiated into endothelial cells and directly participated in tumor angiogenesis through autocrine CCL5 signaling. This intrinsic ability of tumor cells to construct vascular networks may contribute to resistance to VEGF-targeted anti-angiogenic therapies such as bevacizumab.

#### 2.2.3 Chemoresistance

(a) Cellular Quiescence and Immune Evasion

A fraction of OCSCs can reversibly enter a quiescent or slow-cycling state, thereby reducing their vulnerability to cell cycle-dependent cytotoxic agents [90,91,92,93]. This state should not be viewed as passive arrest; rather, it represents a stress-adaptive program that facilitates survival during chemotherapy and provides a reservoir from which residual cells can later re-enter proliferation [92,93,94].

(b) High Expression of Drug Efflux Pumps and Multidrug Resistance

OCSCs may also actively lower intracellular drug exposure through ATP-binding cassette (ABC) transporters, particularly ABCB1 and ABCG2 [95,96,97]. In ovarian cancer, ABCB1/P-gp-mediated efflux is most consistently linked to taxane resistance, whereas the contribution of efflux transporters to platinum resistance appears more context dependent [98,99,100]. Likewise, the side-population phenotype is useful for functional enrichment of high-efflux cells, but it should not be considered synonymous with OCSCs [97,101,102].

(c) Enhanced DNA Damage Repair and Anti-Apoptotic Capacity

Under genotoxic stress, OCSC-enriched models exhibit stronger checkpoint activation and DNA repair signaling than bulk tumor cells [76,103,104,105,106]. In ovarian cancer, ALDH1A1 has been linked to altered regulation of cell-cycle checkpoint and DNA repair networks [76], while recent work further showed that RAD51AP1 supports self-renewal and chemotolerance in CD133^+^ ovarian cancer stem-like cells [107]. These findings support the view that enhanced damage tolerance is a recurrent component of the OCSC phenotype.

(d) The Protective Role of the TME

The drug resistance of OCSCs is further strengthened by the tumor microenvironment [108,109,110]. Hypoxia, inflammatory ascites, and stromal-derived cytokines help maintain stemness and stress tolerance, whereas CAF- and macrophage-derived signals can activate STAT3, NF-κB, and Wnt-associated programs in OCSCs [110,111]. Thus, the TME is best viewed as a source of convergent survival signals rather than merely a passive background.

(e) Metabolic Plasticity and Oxidative Stress Regulation

Metabolic adaptation is another major feature of OCSC chemoresistance [112]. Rather than relying exclusively on a single bioenergetic mode, OCSCs can shift between glycolysis and oxidative phosphorylation according to nutrient availability and treatment pressure [112,113]. In ovarian cancer, platinum exposure can promote mitochondrial OXPHOS programs and enrich ALDH-positive stem-like cells, while OXPHOS inhibition limits this enrichment [112]. Moreover, post-chemotherapy minimal residual disease has been reported to display an adipocyte-like, FAO-dependent state, further underscoring the metabolic flexibility of tumor-initiating cells [114]. ALDH-high cells also exhibit enhanced detoxification of reactive aldehydes and lower ROS levels [115,116,117]. However, the mechanistic interplay between ALDH-mediated aldehyde clearance and NRF2-dependent antioxidant signaling remains to be fully clarified.

## 3 The Multifaceted Role of OCSCs in Ovarian Cancer Progression

### 3.1 Tumor Initiation

The profound lethality of ovarian cancer is fundamentally rooted in its biological behaviors: occult tumor initiation, pervasive peritoneal dissemination, and the frequent chemoresistant relapses. Accumulating reports suggest that OCSCs, a subpopulation with self-renewal and multipotent differentiation potential, play a central role in these malignant processes.

#### 3.1.1 Aberrant Signaling

The perpetuation of the OCSC malignant phenotype is sustained by extensive rewiring of intracellular signaling cascades. However, these pathways are better understood as a hierarchical and interconnected network than as isolated modules. Upstream inputs from the niche, including hypoxia and IL-6, reinforce stemness-associated programs in OCSCs [83,111]. In ovarian cancer spheroids, β-catenin directly regulates ALDH1A1 and supports spheroid integrity and tumorigenicity [118]. Notch3 has been implicated in recurrent disease and platinum resistance in OC [78]. More broadly, Notch-suppressive interventions have been shown to suppress sphere-forming/stemness-associated phenotypes and enhance platinum sensitivity in preclinical OC models [119]. Hedgehog signaling can also contribute to spheroid growth in selected models [120], but current evidence more consistently positions Wnt/β-catenin and Notch3 as central hubs in OCSC maintenance.

Several additional pathways are more appropriately framed as integrative amplifiers of these core programs. IL-6/JAK/STAT3 links inflammatory cues to stemness gene expression, anoikis resistance, and post-chemotherapy OCSC enrichment [111,121]. PI3K/AKT/mTOR and NF-κB cooperate with these inflammatory programs to promote survival, metabolic adaptation, and EMT-associated traits [122,123,124]. Likewise, the Hippo pathway integrates mechanical and transcriptional signals with stemness and chemoresistance, and recent evidence indicates that ARID1A suppresses EMT and stemness of ovarian cancer cells by activating Hippo signaling [125,126].

Recent studies also emphasize that noncanonical Wnt signaling contributes to ovarian cancer dissemination. Tumor-cell ROR2/WNT5A signaling promotes directional migration, mesothelial clearance, and early metastatic seeding [127]. TGF-β signaling is therefore best interpreted not as an isolated pathway but as a regulator of epithelial-mesenchymal plasticity that intersects with Wnt, STAT3, and Hippo/YAP-associated programs [124,128,129]. Overall, a recurring theme is pathway convergence: diverse extracellular and cell-intrinsic cues feed into shared outputs related to stemness maintenance, EMT plasticity, drug tolerance, and metastatic competence, which may help explain the limited durability of single-pathway inhibition [79].

#### 3.1.2 Epigenetic Reprogramming

Unlike the static and irreversible nature of genetic mutations, epigenetic reprogramming endows OCSCs with remarkable phenotypic plasticity [130,131]. This dynamic regulatory mechanism allows OCSCs to flexibly switch between quiescent differentiated state and proliferative stem-like state, serving as a key driver for tumor initiation, the establishment of heterogeneity, and therapeutic resistance [130,132]. The epigenetic landscape of OCSCs is primarily maintained through the coordinated remodeling of DNA methylation, histone modifications, and non-coding RNA networks [133].

First, OCSCs exhibit unique DNA methylation profiles characterized by bidirectional regulation in specific genomic regions [134,135]. On one hand, the aberrant expression of DNA methyltransferases leads to hypermethylation in the promoter regions of tumor suppressor genes and differentiation-related genes, thereby inducing transcriptional silencing and blocking cell differentiation programs. On the other hand, the promoter regions of key transcription factors essential for maintaining stemness remain hypomethylated, ensuring their sustained high-level expression and thus consolidating the stemness features of the cells [136,137,138].

Second, histone modifications and chromatin remodeling serve as gatekeepers for OCSC stemness. EZH2, the core catalytic subunit of PRC2, is markedly overexpressed in OCSCs. It catalyzes H3K27me3 to establish repressive chromatin, silencing developmental regulators like the HOX gene family [139]. Additionally, histone deacetylases induce chromatin compaction by removing acetyl groups, synergistically repressing tumor suppressors and regulating OCSC stemness networks and therapeutic responses [133,140].

Finally, non-coding RNAs finely tune epigenetic mechanisms post-transcriptionally. MicroRNAs and lncRNAs form the core of this regulation [133,141]. For instance, miR-200 family downregulation in OCSCs derepresses ZEB1 and ZEB2, driving EMT and stemness [142,143]. Conversely, lncRNAs such as HOTAIR and H19 act as scaffolds, recruiting chromatin-modifying complexes to specific loci to regulate OCSC phenotypes via local epigenetic alterations [144,145].

#### 3.1.3 Interaction with TME

**Figure 2:**
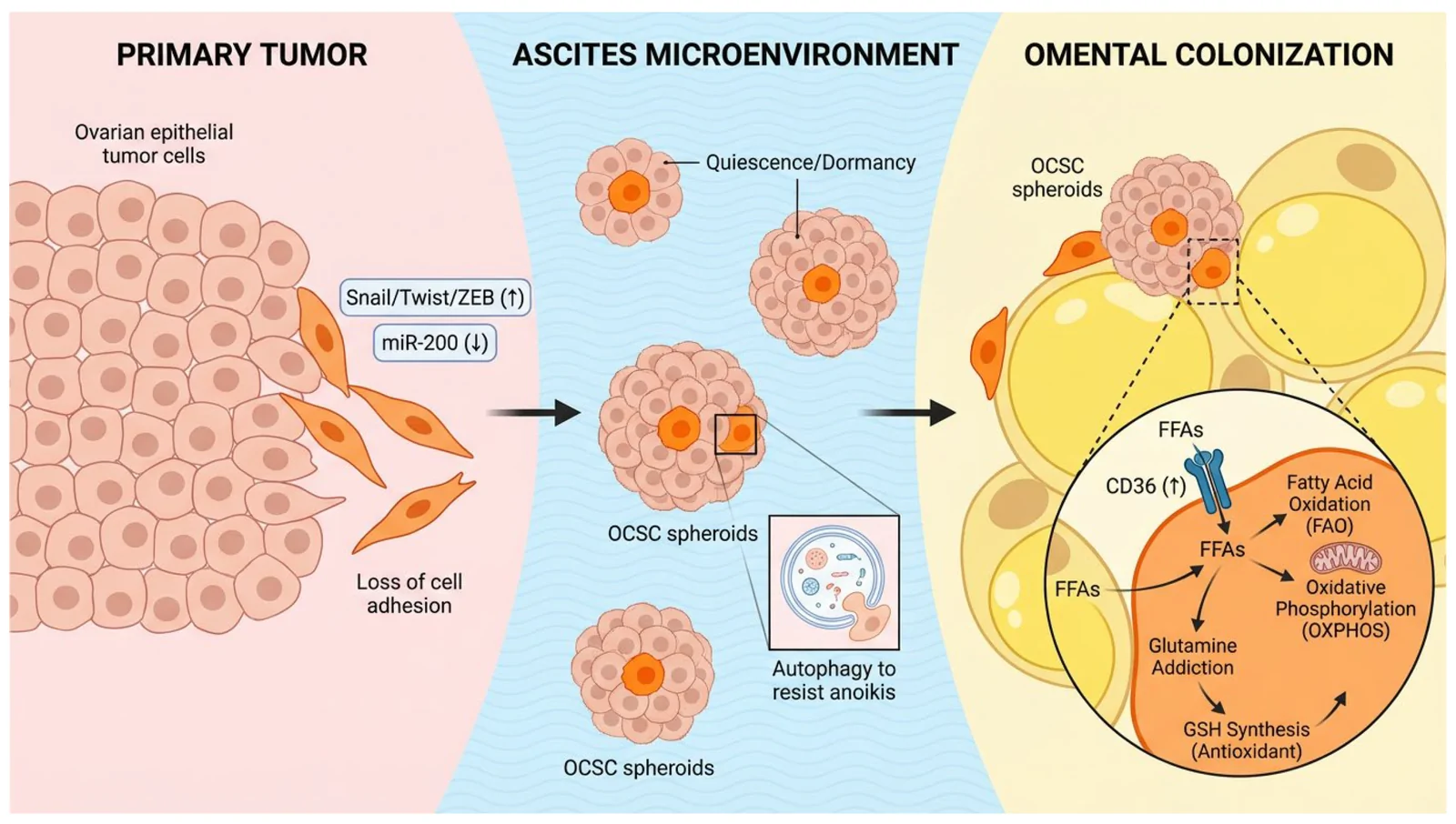
OCSC-driven peritoneal metastasis cascade and metabolic plasticity evolution in OC. The schematic illustrates the stepwise role of OCSCs in intraperitoneal dissemination, including detachment from the primary tumor, survival in the ascites microenvironment, and colonization of the omentum. In the primary tumor, EMT-associated transcriptional programs, including increased Snail/Twist/ZEB signaling and reduced miR-200 expression, promote loss of cell adhesion and acquisition of migratory and stem-like phenotypes. In malignant ascites, detached OCSCs form multicellular spheroids, enter quiescent or dormant states, and activate autophagy-related survival programs to resist anoikis and environmental stress. During omental colonization, adipocyte-rich niches support metabolic reprogramming through CD36-mediated free fatty acid uptake, fatty acid oxidation, oxidative phosphorylation, glutamine addiction, and glutathione synthesis. These adaptive processes collectively promote OCSC survival, metastatic implantation, chemoresistance, and tumor recurrence.

As shown in Fig. 2, OCSC-driven peritoneal dissemination involves detachment from the primary tumor, survival in the ascites microenvironment, and colonization of the omentum, accompanied by dynamic metabolic adaptation. The TME’s cellular and non-cellular components provide an essential niche for tumor initiation and early colonization via precise paracrine networks and physical interactions [146,147].

CAFs support OCSC stemness through paracrine cytokine signaling, including IL-8-associated stemness programs and IL-6/JAK2/STAT3 activation [28,85]. Beyond CAF-derived cytokine signaling, IL-6-related pathways also support ovarian cancer cell survival during dissemination. In ovarian clear cell carcinoma, an autocrine IL-6-SPINK1 axis promotes anoikis resistance and metastatic spread [121]. Furthermore, Wnt/β-catenin signaling is widely recognized as an important pathway in maintaining OCSC phenotypes [148].

At the immune microenvironment level, TAMs and OCSCs establish a reciprocal symbiosis that serves tumorigenesis [149]. OCSCs secrete CSF-1 to recruit and polarize monocytes into M2 TAMs, which in turn release TGF-β, EGF, and IL-10 [149,150,151]. These factors build an immunosuppressive barrier against cytotoxic T cells and directly stimulate OCSC proliferation, accelerating macroscopic tumor formation [84,152]. Physical TME remodeling is equally vital. Rapid tumor growth causes severe local hypoxia [153,154], where hypoxia-inducible factors (HIF-1α and HIF-2α) activate stemness genes. HIF-2α directly upregulates OCT4, while HIF-1α synergizes with NICD to enhance Notch target transcription, maintaining the undifferentiated state [155,156]. Concurrently, hypoxia-induced lysyl oxidase increases ECM crosslinking and stiffness. This physical cue triggers integrin-mediated mechanotransduction, further amplifying the invasive potential of tumor cells [154,157].

The ascites microenvironment, which is characteristic of advanced ovarian cancer, constitutes a distinct fluid microenvironment for intraperitoneal dissemination [147]. Bioactive lipids, free fatty acids, and exfoliated stromal cells enriched in ascites provide crucial metabolic substrates and survival signals for suspended tumor cells. They also promote the aggregation of cells into multicellular spheroids under anchorage-independent conditions [153,158,159]. Served as the basic functional units of intraperitoneal dissemination, these spheroid structures not only maintain high levels of stemness signals but also possess extremely strong capabilities for intraperitoneal adhesion and colonization [159,160].

### 3.2 Metastasis

The unique intraperitoneal dissemination pattern of ovarian cancer is closely related to the characteristics of OCSCs. OCSCs not only have the ability to detach from the primary lesion but can also survive in the harsh peritoneal environment and colonize distant organs.

#### 3.2.1 Epithelial-Mesenchymal Transition

EMT is a fundamental biological program in embryonic development and cancer progression [161]. In OC, EMT-associated plasticity is closely linked to invasive behavior, stem-like traits, and intraperitoneal dissemination [162,163]. During this complex cellular reprogramming process, OCSCs undergo dramatic morphological remodeling, accompanied by the loss of apical-basal polarity and the dissociation of intercellular tight junctions, thereby acquiring high motility [161,164]. Characteristic changes at the molecular level are primarily manifested by the significant suppression of epithelial markers such as E-cadherin, Claudins, and Occludins, along with the compensatory upregulation of mesenchymal markers [63,164]. This classic cadherin switch phenomenon weakens homotypic intercellular adhesion and promotes the anoikis resistance of tumor cells, enabling them to detach from the primary tumor and colonize the peritoneum or distant organs [62].

Notably, there is a tight reciprocal regulatory mechanism between the EMT program and the acquisition of tumor stemness [165]. Current evidence indicates that activation of the EMT program can directly induce the acquisition of stem cell-like characteristics. This process is regulated by core transcription factor families (EMT-TFs), including the Snail, Twist, and ZEB families. These transcription factors can remodel stemness-related transcriptional programs through transcriptional and epigenetic mechanisms. The most classic mechanism is Snail binding to the E-box sequence in the E-cadherin promoter region to directly repress its transcription, thereby initiating the EMT process [63,166]. Within the EMT-TF regulatory network, the miR-200 family and ZEB1/2 form a classic double-negative feedback loop: miR-200 maintains the epithelial phenotype by inhibiting ZEB1/2, while ZEB1/2 can reversely inhibit miR-200 to promote EMT. Therefore, downregulation of miR-200 is often accompanied by enhanced EMT and the acquisition of CSC-like or undifferentiated phenotypes [167,168,169,170].

Furthermore, histological and metastasis-related studies suggest that ovarian cancer exhibits significant phenotypic heterogeneity at different spatial sites. A subpopulation of tumor cells with a more EMT/mesenchymal-biased phenotype can be observed at the invasive front of metastatic lesions. Meanwhile, the enrichment of CD44 variant-positive cells can be observed in peritoneal disseminated lesions, which is associated with metastasis initiation and drug resistance. Combined with functional evidence of OCSC marker subpopulations, such as CD44^+^CD117^+^ or ALDH1^+^, in tumorigenicity and therapeutic resistance, this suggests that EMT-related plasticity may link between the metastatic cascade and stemness maintenance [79,80,171].

#### 3.2.2 Quiescence and Dormancy

The bottleneck in ovarian cancer treatment is the high recurrence rate following chemotherapy, the core mechanism of which is attributed to the unique quiescence and dormancy characteristics of OCSCs [79,166]. In the face of cytotoxic attacks from paclitaxel and platinum-based drugs, OCSCs can enter a reversible G0 phase cell cycle arrest, thereby naturally evading conventional therapies that target rapidly proliferating cells. This adaptive resistance mechanism allows minimal residual disease to remain latent in the body for a long time, becoming the root cause of future tumor recurrence [167,168,170].

The dormancy of OCSCs is not a passive stagnation but a survival strategy actively regulated by complex molecular networks [172]. Studies have confirmed that autophagy plays a key role in this process. For instance, the re-expression of the tumor suppressor gene DIRAS3 can induce autophagy by inhibiting the PI3K/AKT/mTOR pathway, providing metabolic substrates for dormant cells and clearing oxidative stress damage [173,174,175]. Meanwhile, the upregulation of specific kinases such as DYRK1A and the alteration of the p38 MAPK/ERK signaling ratio synergistically regulate cell cycle exit and maintain the expression of stemness transcription factors, ensuring that cells retain their tumorigenic potential during dormancy [176,177]. Furthermore, the dynamic interaction between dormant OCSCs and the tumor microenvironment is the foundation for their long-term survival [94]. Hypoxic environments and stromal cell-secreted factors, such as GAS6-AXL, not only induce dormancy but also inhibit MHC-I antigen presentation in tumor cells, weakening T cell recognition and clearance, thereby promoting immunosuppression to evade immune surveillance [178,179].

Thereby, the plasticity to switch between dormancy and proliferation provides OCSCs a survival advantage in the metastatic cascade, allowing them to overcome spatiotemporal barriers, persist in distant organs, and drive metastasis.

#### 3.2.3 Metabolic Plasticity

During metastasis and recurrence, OCSC metabolic plasticity is a key adaptation to microenvironmental and chemotherapeutic stress [180]. Unlike most proliferating tumor cells that rely on aerobic glycolysis, OCSCs exhibit metabolic flexibility. They often favor OXPHOS and maintain stem-like traits and survival during glucose deprivation [181,182]. Ovarian cancer cells display marked metabolic heterogeneity between glycolysis and OXPHOS; chemoresistant lines are highly active and can switch between these pathways [183]. Platinum chemotherapy upregulates OXPHOS programs and enriches ALDH-positive stem-like cells, whereas OXPHOS inhibition reduces this post-treatment enrichment [112].

In the peritoneal metastatic niche, interactions between OCSCs and omental adipose tissue drive metabolic reprogramming [184,185]. The hypoxic and nutrient-deprived ascites microenvironment forces tumor cells to utilize lipids when glucose is limited [186]. Omental adipocytes supply lipids and upregulate CD36 on tumor cells, enhancing free fatty acid uptake. This axis provides a metabolic survival advantage for stem-like, tumor-initiating cells under stress [187]. Consequently, CPT1A and fatty acid oxidation are upregulated in post-chemotherapy and platinum-resistant models, allowing tumor cells to meet energy demands via mitochondrial *β*-oxidation of long-chain fatty acids. Inhibiting CPT1A/FAO enhances chemosensitivity and mitigates resistance across models [114].

Mechanistically, FAO boosts ATP via oxidative phosphorylation for survival under stress and synergizes with NADPH pathways to maintain redox homeostasis [188,189]. This NADPH-dependent system buffers chemotherapy-induced ROS, preventing apoptosis [190,191]. Consequently, may preferentially survive chemotherapy and become enriched after treatment, thereby contributing to the persistence of minimal residual disease [114].

Additionally, altered amino acid metabolism is vital for OCSC stemness and recurrence [192]. Metabolomics reveal a distinct glutamine addiction in OCSCs [193]. Glutamine replenishes the TCA cycle for biosynthesis and acts as a glutathione precursor, bolstering oxidative stress tolerance [194,195]. Ultimately, by integrating FAO and glutamine metabolism, OCSCs form a flexible metabolic defense against starvation and chemotherapy, ensuring long-term survival and providing the energy and materials for eventual relapse.

### 3.3 Recurrence and Resistance

Despite standard cytoreductive surgery and platinum/paclitaxel chemotherapy [196], most ovarian cancer patients eventually relapse [3,8]. The following sections therefore focus on three recurrent resistance modules implicated in OCSCs: drug efflux, enhanced DNA damage repair, and redox-detoxification programs [97,197,198,199].

#### 3.3.1 Drug Efflux Pumps

A well-established mechanism of OCSC chemoresistance is the overexpression or enhanced function of ATP-binding cassette (ABC) transporters, such as P-glycoprotein (P-gp) and breast cancer resistance protein (BCRP). These transporters utilize ATP hydrolysis to actively extrude chemotherapeutics, reducing intracellular drug levels and promoting survival [200,201]. In ovarian cancer models, ABCB1/P-gp-mediated efflux correlates consistently with taxane resistance; its role in resistance to other drugs varies depending on the cellular context and experimental setup [98,99,100].

In flow cytometry, cells that rapidly efflux Hoechst 33342 dye form SP, a phenotype used to enrich stem-like cells with high efflux capacity [202]. ABCG2 is a key molecular determinant of this phenotype [102]. While ovarian cancer SP cells show enhanced tumorigenicity and chemoresistance, making them useful for OCSC enrichment, the SP is highly heterogeneous. The specific transporters driving the SP phenotype vary among cell lines. Therefore, SP should be considered a functional enrichment phenotype rather than synonymous with OCSCs [97].

Clinically, genomic and transcriptomic studies indicate that abnormal ABCB1 activation occurs in recurrent and resistant ovarian cancers, driven by chemotherapy selection pressure [203]. Conversely, evidence for consistent ABCG2 upregulation in recurrent versus primary tumors remains mixed, likely due to variations in cohorts, subtypes, and prior treatments [100]. Ultimately, drug efflux mechanisms enable stem-like cells to survive chemotherapy and exhibit multidrug resistance, preventing complete eradication by single-agent regimens. Within the context of MRD, these mechanisms are considered critical drivers of recurrence [204].

#### 3.3.2 Enhanced DDR

Platinum cytotoxicity primarily stems from DNA adducts and crosslinks, which distort DNA conformation, block replication/transcription, and trigger DDR and apoptosis [104,105]. Studies utilizing OCSC-enriched models or subpopulations defined by markers including CD133 and CD117 indicate that under treatment-induced DNA damage and replication stress, OCSCs increasingly rely on ATR/CHK1 checkpoints and homologous recombination (HR) repair to survive and establish residual disease [106,205,206]. For instance, Wen et al. showed that EZH2 promotes CHK1 signaling to maintain OCSC stemness and chemoresistance [207]. Similarly, Bellio et al. found that PARP inhibitors enrich CD133^+^/CD117^+^ cells, inducing G2/M arrest and dynamic RAD51 foci changes. This indicates a robust DNA repair capacity in these cells, which blunts the synthetic lethality of PARP inhibitors and increases recurrence risk [205].

Studies on platinum-resistant ovarian cancer indicate that enhanced nucleotide excision repair, such as upregulated ERCC1-XPF, promotes the removal of platinum-DNA adducts and blunts drug efficacy. However, while this highlights a general resistance mechanism, its specificity to OCSCs requires further controlled validation [208,209,210]. In the context of BRCA mutations/HRD, longitudinal genomic profiling reveals diverse evolutionary trajectories, suggesting that recurrence is driven by clonal selection and adaptive evolution under therapeutic pressure [203,206]. Collectively, an augmented and adaptive DNA repair network serves as a core survival strategy for OCSCs, driving treatment failure and relapse.

#### 3.3.3 A Coordinated Redox-Detoxification Defense System

OCSC chemoresistance is increasingly understood as the result of multiple coordinated defense mechanisms rather than a single process [75,113,211]. Beyond physical drug efflux, ALDH-mediated metabolic reprogramming forms a critical defense against cytotoxicity [75,212]. Isozymes like ALDH1A1 are not merely stemness markers but functional metabolic detoxifiers [52,132]. Platinum-induced stress increases lipid peroxidation and the accumulation of reactive aldehydes such as 4-HNE. High ALDH expression oxidizes these reactive aldehydes into less toxic carboxylic acids, reducing electrophilic stress and enhancing chemoresistance [116,117,213]. This enzymatic detoxification may also contribute to redox homeostasis and help to buffer oxidative stress in OCSC-enriched settings [117].

Compared with bulk tumor cells, OCSC-enriched models have been reported to maintain lower ROS levels, potentially through at least two processes: enhanced ALDH-mediated aldehyde detoxification and NRF2-associated antioxidant defenses linked to stemness and resistance [214,215,216,217].

Evidence from ALDH-high ovarian CSC models further suggests a functional ALDH1A1–p62–NRF2 circuit, as ALDH1A1 silencing reduces NRF2, p62, and CSC-associated markers, whereas NRF2 knockdown suppresses chemoresistance, colony/sphere formation, and tumor growth [198,214,217]. Prudently, ALDH detoxification and NRF2 antioxidant programs likely act in parallel or compensatorily to retain OCSCs in a low oxidative-stress state [217]. Importantly, emerging evidence suggests that redox-buffering and lipid peroxide detoxification programs may also influence ferroptosis sensitivity in therapy-persistent, stem-like ovarian cancer cells. Platinum-tolerant ovarian cancer cells with partial stem-like features depend on an FZD7–β-catenin–Tp63–GPX4 pathway for survival and display heightened sensitivity to GPX4 inhibition, supporting the existence of a targetable ferroptotic vulnerability in therapy-persistent stem-like populations [218]. Likewise, platinum treatment increases mitochondrial activity and enriches ALDH^+^ OCSCs, whereas OXPHOS inhibition blocks this enrichment *in vitro* and *in vivo*, linking therapy-induced metabolic rewiring to the persistence of residual stem-like cells and raising the possibility of altered ferroptosis sensitivity in these populations [112]. Consistently, NRF2 expression rises during ovarian cancer spheroid formation together with GPX4, and NRF2 suppression enhances sensitivity to GPX4 inhibitors while reducing spheroid growth, further supporting cooperation between antioxidant and anti-ferroptotic effects [219]. Thus, by limiting the accumulation of lipid peroxides and sustaining GPX4-centered detoxification, OCSCs may raise the ferroptosis threshold and blunt therapy-induced death [192,220,221]. By contrast, evidence connecting cuproptosis to bona fide OCSCs remains preliminary. Because canonical cuproptosis preferentially affects respiration-dependent cells through copper binding to lipoylated TCA-cycle proteins [222], the OXPHOS-high state of post-treatment OCSCs suggests a plausible—though still inferential—metabolic vulnerability [112,222].

Supporting this hypothesis, anisomycin inhibited human CD44^+^/CD133^+^ OCSCs and downregulated YY1-controlled lipoic acid pathway genes, including FDX1, DLD, DLAT, and PDHB, suggesting potential cuproptosis induction [223]. However, recent ovarian cancer studies indicate that FDX1 can also promote paclitaxel resistance via copper-dependent ULK1/ATG13-mediated autophagy, underscoring the context-dependent role of copper metabolism in OC [224]. Therefore, cuproptosis should currently be framed as a promising but still unconfirmed therapeutic vulnerability in OCSCs rather than an established mechanism of OCSC chemoresistance.

Beyond supporting ferroptosis resistance, this low-ROS state may also reduce the susceptibility of OCSCs to mitochondria-mediated apoptosis [225,226]. Because oxidative stress triggers mitochondrial permeabilization and Caspase activation, ALDH-mediated aldehyde clearance and redox maintenance prevent OCSCs from reaching the apoptotic tipping point, even during chemotherapy [227]. Thus, ALDH is a functional survival driver, not just a static marker. By integrating detoxification, maintaining redox homeostasis, and suppressing stress-induced death pathways, ALDH equips OCSCs to adapt to toxic environments. Alongside drug efflux, these metabolic and antioxidant defenses incline to play a role in tumor recurrence and refractoriness.

## 4 Therapeutic Strategies Targeting Cancer Stem Cells

### 4.1 Targeting Stem Cell-Specific Signaling Pathways

OCSCs depend on multiple stemness pathways for maintenance and resistance, including Notch, Wnt, JAK2/STAT3, PI3K/PTEN/AKT, Hippo-YAP, NF-κB, and Hedgehog. In ovarian cancer, Notch signaling is notably disease-specific. Notch3 is frequently amplified or overexpressed in high-grade serous ovarian cancer and correlates with poor prognosis, indicating its role in sustaining tumor stemness and survival [228,229,230]. Functionally, preclinical studies indicate that Notch-suppressive interventions can reduce stem-like cell populations and enhance platinum sensitivity in ovarian cancer models, supporting their potential as a chemosensitizing strategy [119]. Similarly, Wnt/β-catenin drives tumorsphere formation, metastasis, and chemoresistance. Its downstream targets upregulate drug efflux transporters and stress adaptation, facilitating the survival and expansion of resistant clones under taxane and platinum pressure [148,231,232]. These findings further underscore the role of stemness-associated signaling in OCSC maintenance and therapy resistance.

The JAK2/STAT3 axis is implicated in ovarian cancer treatment response. Patient-derived acellular ascites fluid can induce STAT3 activation and reduce sensitivity to standard-of-care drugs in ovarian cancer cell lines. In addition, experimental silencing of STAT3 reverses inherent and induced chemoresistance in ovarian cancer cells, supporting STAT3 as a potential therapeutic target [233,234].

Additionally, pathways like PI3K/PTEN/AKT, Hippo/YAP, NF-κB, and Hedgehog may further reinforce stemness- and therapy resistance-associated signaling networks. Crosstalk and compensatory bypass among these pathways may contribute to the limited and variable efficacy of single-target inhibition in heterogeneous tumors [124,235,236]. Therefore, a more rational translational strategy may involve patient stratification based on pathway activation markers and the integration of combination or sequential therapies into standard regimens to reduce tumor burden, target residual stem-like cells, and delay relapse.

Beyond developmental pathways, targeting stem cell robustness is gaining traction [235]. OCSCs typically enter a slow-cycling or quiescent state to evade cell cycle-dependent therapies, resisting stress via enhanced redox homeostasis and drug efflux [232]. For instance, the CD44v/xCT axis promotes cysteine uptake and GSH synthesis, enhancing ROS scavenging to protect stem-like cells from stress-induced death. This mechanism provides a rationale for combining chemotherapy with redox-targeted agents to dismantle the resistance foundation in ovarian cancer [237,238]. Ultimately, integrating stemness pathway inhibition with robustness-targeted interventions, tailored to the tumor microenvironment and treatment stage, represents a more viable translatable strategy for targeting OCSCs.

Translationally, the IL-6/JAK/STAT3 axis has already entered ovarian cancer clinical trials. In the NRG Oncology phase I/II study NCT02713386, the JAK1/2 inhibitor ruxolitinib was combined with neoadjuvant paclitaxel/carboplatin, phase I enrolled 17 patients and phase II randomized 130 patients, with a median progression-free survival of 14.6 versus 11.6 months (HR = 0.702, P = 0.059), although grade 3/4 anemia, neutropenia, and thromboembolic events were more frequent in the ruxolitinib arm [239]. In parallel, the stemness/STAT3-targeting agent napabucasin (BBI608) has been evaluated in combination with weekly paclitaxel in advanced malignancies, including a platinum-resistant/refractory ovarian, fallopian tube, or primary peritoneal cancer cohort (NCT01325441), however, based on currently accessible primary reports, the ovarian-specific clinical evidence remains at early phase [240]. These studies support a clinically actionable framework in which STAT3-pathway activation markers are used to enrich for patients most likely to benefit from pathway blockade rather than applying CSC-directed agents indiscriminately [239,241]. This interpretation is further supported by the limited clinical activity of other stemness-related agents in ovarian cancer: RO4929097 produced no objective responses in 40 evaluable platinum-resistant cases, whereas vismodegib failed to demonstrate meaningful clinical benefit in 104 randomized patients, with Hedgehog ligand expression detected in only 13.5% of archival tissues [242,243]. Together, these findings highlight the limitations of testing stemness-directed therapies in unselected populations. Representative clinical studies of stemness-related pathway inhibitors in ovarian cancer are summarized in Table 2.

**Table 2:** Representative clinical studies of stemness-related pathway inhibitors in ovarian cancer.

Pathway	Agent	Trial	Phase	Population	Key Findings	Evidence Level
JAK1/2–STAT3	Ruxolitinib	NCT02713386	Phase I/randomized Phase II	Advanced ovarian/fallopian tube/primary peritoneal carcinoma	Phase II enrolled 130 patients; median PFS 14.6 vs. 11.6 months, HR 0.702, P = 0.059; higher grade 3–4 anemia, neutropenia, and thromboembolic events	Peer-reviewed randomized data; signal of activity, but not definitive benefit
Stemness-related/STAT3-associated	Napabucasin	NCT01325441	Phase Ib/II	Protocol-eligible platinum-resistant/refractory epithelial ovarian/fallopian tube/primary peritoneal cancer	Early ovarian-cancer clinical exploration reported	Registry/protocol + conference-level evidence; exploratory
Notch	RO4929097	Single-agent study in recurrent platinum-resistant EOC	Phase II	Recurrent platinum-resistant epithelial ovarian cancer	45 enrolled, 40 evaluable; no objective responses; median PFS 1.3 months	Peer-reviewed negative phase II study
Hedgehog	Vismodegib	Maintenance therapy in second/third CR	Randomized Phase II	Recurrent epithelial ovarian/fallopian tube/primary peritoneal cancer in second or third CR	104 randomized; median PFS 7.5 vs. 5.8 months (HR 0.79); Hedgehog ligand expression in 13.5% of archival tissues	Peer-reviewed randomized phase II data; prespecified benefit not achieved

CR, complete remission; EOC, epithelial ovarian cancer; HR, hazard ratio; PFS, progression-free survival.

### 4.2 Nanoparticle-Based Drug Delivery Systems

Despite the growing list of CSC-targeted candidates, clinical translation is often hindered by poor drug delivery and tissue exposure [244,245,246]. CSCs typically reside in specific niches—such as hypoxic, perivascular, or stroma-rich regions—characterized by abnormal perfusion, elevated interstitial pressure, and metabolic gradients. This causes uneven drug distribution, preventing *in vitro*-effective drugs from reaching sufficient *in vivo* concentrations at CSC sites [244,245]. Thus, nanoparticle-based or ligand-mediated delivery systems are essential to bridge the gap between target identification and drug accessibility.

In OCSC research, the hyaluronic acid (HA) receptor CD44 is a prime example [247]. Strategies exploiting the high HA-CD44 affinity, such as HA-drug conjugates or HA-coated nanoparticles, enhance selective uptake by CD44^+^ cells. For peritoneal dissemination in ovarian cancer, intraperitoneal administration further minimizes systemic toxicity [248]. This approach crucially shifts CSC markers from mere prognostic tools to actionable delivery portals, widening the therapeutic window against resistant subpopulations [248,249]. Similarly, TIC studies highlight the viability of targeting CSC surface receptors for drug or immunomodulator delivery, indicating that a marker-delivery-killing loop is broadly applicable across solid tumors [249,250].

However, delivery systems face challenges from CSC heterogeneity and non-specific marker expression. Common markers (e.g., CD44, CD133, EpCAM, ALDH) are also expressed in normal or non-CSC tumor cells and are dynamically regulated by therapeutic stress, hypoxia, and inflammation, causing inadequate coverage and off-target toxicity [251,252,253,254,255]. Consequently, future systems tend to evolve from single-ligand targeting to multi-ligand or logic-gated recognition, delivering combinatorial payloads of chemotherapeutics and metabolic, immune, or epigenetic modulators to counteract CSC state transitions. Ultimately, nanodelivery must serve as a platform for combined mechanistic interventions to target diverse CSC subpopulations and states simultaneously.

### 4.3 Combination Therapies

First-line systemic treatment for ovarian cancer relies on platinum-taxane chemotherapy, yielding high initial responses. Clinical practice now integrates this with targeted therapies, immunotherapies, anti-angiogenics, or PARP inhibitor maintenance to prolong remission and improve outcomes [196]. Despite high initial response rates, recurrence and resistance remain pervasive. Mechanistic studies indicate that platinum therapy enriches ALDH^+^ stem-like cells in residual lesions, driving subsequent relapse and chemoresistance [256,257,258].

On the other hand, chemotherapy itself possesses immunomodulatory effects and can remodel the TME, providing a window of antigen release and inflammation initiation for immunotherapy. However, it may also cause damage to immune cells. Especially under high-dose regimens, it can lead to a decrease in the number of T, B, and NK cells, thereby weakening the synergy of immunotherapy and potentially promoting recurrence [259,260]. Therefore, the key to chemotherapy combination strategies lies in ensuring the control of the main tumor bulk while avoiding excessive immunosuppression as much as possible, and addressing the residual cell populations with the highest recurrence potential through CSC-targeted drugs or immunomodulators.

### 4.4 AI-Guided Drug Repurposing

*In silico* drug repurposing has been applied to OCSCs by integrating OCSC-enriched transcriptomic signatures with drug-perturbation resources. Huang and colleagues derived an OCSC-associated expression signature from CSC-enriched ovarian cancer cell populations and queried the Connectivity Map (CMap), together with CO-eXpression ExtrapolatioN (COXEN)-based coexpression extrapolation, to prioritize compounds predicted to reverse the OCSC signature, nominating disulfiram among the candidate small molecules [261,262]. Functional studies further reported that disulfiram, with or without copper ions, inhibited ALDH activity, depleted ALDH-positive stem-like cells, suppressed tumorsphere formation and clonogenic growth, and enhanced cisplatin-induced apoptosis; these effects were accompanied by increased intracellular ROS, consistent with redox-related mechanisms [215]. More recent AI studies have expanded this framework from transcriptomic signature reversal to machine-learning-based therapy-response prediction and candidate prioritization in ovarian cancer. For example, gene expression-based models using feature selection with random forest and support vector machine classifiers predicted platinum–paclitaxel and platinum-only response in serous ovarian cancer [263], whereas a 2025 single-cell and multi-omics-based CSOARG model estimated lower IC50 values in low-risk tumors for cisplatin, 5-FU, camptothecin, gefitinib, nilotinib, bosutinib, selumetinib, dabrafenib, and temozolomide, thereby providing a complementary route for prioritizing candidate agents for further testing [264].

Machine learning-derived stemness indices provide a complementary strategy to quantify dedifferentiation programs and guide compound prioritization. Malta and colleagues developed the mRNA expression-based stemness index (mRNAsi) using one-class logistic regression (OCLR) trained on pluripotent stem cell profiles and their differentiated progeny, enabling transcriptome-based stemness scoring across tumor types [265]. In ovarian cancer, mRNAsi-linked coexpression modules have been queried against perturbational resources such as CMap to nominate compounds predicted to attenuate stemness-associated programs [266]. More broadly, recent integrative AI frameworks indicate that molecular and immune features can be incorporated upstream of therapeutic prioritization: in a 2025 multi-omics study, machine learning models combining genomic and immune features outperformed single-parameter models for therapy-response prediction, with early NK-cell abundance and TP53 status emerging as key determinants in HGSOC [267]. In parallel, an AI-driven multi-omics framework integrated TCGA, GDSC, and validation cohorts using variational autoencoders, LSTM networks, and multitask MLPs for molecular subtyping, survival analysis, and IC50 prediction, and coupled these outputs with structure-guided drug repurposing to identify lactylation-associated therapeutic vulnerabilities linked to LDHA/SLC16A3 and cisplatin resistance [268]. These *in silico* predictions require subsequent validation in OCSC-focused functional assays, including the ALDH-positive fraction, tumorsphere formation, and tumor-initiating capacity [215].

### 4.5 Current Limitations and Future Challenges of Targeted Therapies

Although targeted therapies against OCSCs have made significant progress in theoretical and preclinical models, their clinical translation still faces severe challenges. First, the off-target toxicity of targeted drugs is a major limiting factor. Since pathways like Notch and Wnt also play critical roles in maintaining the homeostasis of normal tissue stem cells, systemic inhibition may lead to severe side effects, limiting the therapeutic window of the drugs. Second, the plasticity of tumor cells makes treatment more complex. Studies have found that under chemotherapy pressure, non-stem tumor cells can regain stem cell properties through a dedifferentiation process, which means that merely eliminating existing OCSCs may not be sufficient to completely prevent recurrence. Finally, OCSCs themselves exhibit high heterogeneity. Different stem cell subpopulations in different patients or even within the same tumor may rely on different driving mechanisms. The lack of broad-spectrum specific markers makes precise stratified treatment difficult to achieve. Future research needs to focus on resolving heterogeneity at the single-cell level and developing dynamic monitoring technologies, in order to design more precise comprehensive treatment regimens with controllable side effects.

## 5 Conclusion

In summary, OCSCs are key drivers of tumor initiation, metastasis, therapeutic resistance, and recurrence in OC. Their biological functions are sustained by stemness-associated signaling, cellular plasticity, metabolic adaptation, and dynamic interactions with the tumor microenvironment, making them important contributors to disease progression and poor clinical outcomes. Although substantial progress has been made in understanding OCSC biology and developing targeted strategies, major challenges remain, including tumor heterogeneity, phenotypic plasticity, and limited clinical translation. Therefore, a deeper mechanistic understanding of OCSCs will be critical for overcoming current translational barriers and improving long-term therapeutic outcomes in OC.

## Data Availability

Not applicable.
